# Assessment of the responsiveness of a public health service from the perspective of older adults

**DOI:** 10.1590/S1518-8787.2017051006854

**Published:** 2017-06-20

**Authors:** Denise da Silva Melo, René Duarte Martins, Renata Patrícia Freitas Soares de Jesus, Isabella Chagas Samico, Antônio Carlos Gomes do Espírito Santo

**Affiliations:** I Programa de Pós-Graduação em Saúde Humana e Meio Ambiente. Centro Acadêmico de Vitória. Universidade Federal de Pernambuco. Vitória de Santo Antão, PE, Brasil; II Núcleo de Saúde Coletiva. Centro Acadêmico de Vitória. Universidade Federal de Pernambuco. Vitória de Santo Antão, PE, Brasil; III Grupo de Estudos de Gestão e Avaliação em Saúde. Diretoria de Pesquisa. Instituto de Medicina Integral Prof. Fernando Figueira. Recife, PE, Brasil; IV Programa de Pós-Graduação em Gerontologia. Universidade Federal de Pernambuco. Recife, PE, Brasil

**Keywords:** Aged, Health Services for the Aged, Health Services Evaluation

## Abstract

**OBJECTIVE:**

To assess the quality of health care of older adults using as a parameter the assessment of the responsiveness of the service.

**METHODS:**

This is a descriptive cross-sectional study conducted in a reference unit of the Brazilian Unified Health System at the outpatient level. The sample was probabilistic and had 385 older adults; data collection occurred in 2014. The domains assessed were: choice, autonomy, confidentiality, dignity, communication, physical facilities, and fast service. To this end, we used Pearson correlation test and Fisher’s exact test.

**RESULTS:**

The domains of dignity, confidentiality, and communication reached the highest level of adequate responsiveness. On the other hand, freedom of choice and fast service received the worst assessments. Participation in decision-making regarding treatment was significantly lower among the older adults who had no education. In addition, the older adults that self-reported as black receive a lower quality of care regarding clear explanation and respected privacy in the appointment, when compared to users of any other race.

**CONCLUSIONS:**

Although most domains studied have receive a positive assessment, we have found a need for an equal care by the health professionals, regardless of race, education level, or any other adjective characteristic of older adults, users of public health services.

## RESUMO

**OBJETIVO:**

Avaliar a qualidade da atenção à saúde da população idosa usando como parâmetro a avaliação da responsividade do serviço.

**MÉTODOS:**

Trata-se de um estudo descritivo, de corte transversal, realizado em uma unidade de referência do Sistema Único de Saúde em nível ambulatorial. A amostra foi probabilística composta por 385 idosos e a coleta de dados ocorreu em 2014. Foram avaliados os domínios: escolha, autonomia, confidencialidade, dignidade, comunicação, instalações físicas e atendimento rápido. Para tanto, foram utilizados o teste de correlação de Pearson e o teste de Fisher.

**RESULTADOS:**

Os domínios dignidade, confidencialidade e comunicação atingiram o maior nível de responsividade adequada. Por outro lado, a liberdade de escolha e o atendimento rápido receberam as piores avaliações. A participação na tomada de decisões a respeito do tratamento foi significativamente menor entre os idosos que não frequentaram a escola. Além disso, os idosos que se autodeclararam negros receberam um atendimento de menor qualidade no que diz respeito à explicação clara e a privacidade respeitada mediante consulta, quando comparados aos usuários de outra raça.

**CONCLUSÕES:**

Apesar de a maioria dos domínios estudados receberem uma avaliação positiva, evidenciou-se a necessidade de um atendimento igualitário por parte dos profissionais de saúde, independentemente de raça, nível de escolaridade ou qualquer outra característica adjetiva referente aos usuários idosos atendidos nos serviços de saúde públicos.

## INTRODUCTION

The growth of the population of older adults is a worldwide phenomenon, and it happens in an accelerated and significant way in Brazil^21^. It is estimated that in 2025 the country will be the sixth in the world in absolute number of older adults, amounting to 33,800,000 individuals, going from the current 2.7% to 14.7% of the general population^10^. This fact results in deep changes in society and new demands of health, as the demographic transition at the same time as the epidemiological transition involve the greater use of health services because of the exacerbation of chronic conditions, increased frequency of comorbidities, and higher incidence of functional decline^13,14^.

The health system needs to meet adequately the growing demands of the aging population, taking into account issues that reflect the peculiarities of that age group. That has brought up the need for the elaboration and adequacy of public policies and actions to improve the living conditions of the new population profile.

Thus, social policies were created and scaled in Brazil focused on older adults, especially in health, namely: the National Policy of Social Assistance, the Statute of the Older Adult, the International Plan of Action on Aging, and the National Health Policy for Older Adults, among others. All recognize the need for the consolidation of a social protection network for older adults, towards a society for all ages. However, there are several difficulties to implement them, such as: poor allocation of resources, the fragile information system for the analysis of living and health conditions, and the inadequate training of human resources^5,6^.

Thus, the assessment of these services is considered an essential part in the planning and management of the health system, considering that their improvement is achieved when we have the satisfaction of users as a goal^7,15^.

The incorporation of research methods of customer satisfaction for assessment studies has allowed the identification of areas that can be improved in the care provided by health services, and they have become an important tool for the management of the area^1^.

However, studies have shown that different individual expectations, influenced by different sociocultural realities, lead to a subjective bias when we assess the services from the point of view of users^7,9^. Given that, the search for greater objectivity in these studies has culminated in the development of a new concept – responsiveness – by the World Health Organization (WHO)^28^.

This concept refers to the way the system works in relation to the legitimate expectations of users in relation to non-medical aspects of care, i.e. the elements not directly linked to the state of health, but that may influence it^9^.

A study conducted by the WHO has noted that approximately 8% of patients who assessed the care provided at the outpatient level indicated low responsiveness of the health services^28^. In Brazil, studies conducted at different levels of social care (hospitals and primary health care) have shown that dignity, confidentiality, and communication between professional and patient were the most well-respected aspects, in line with findings in other countries^2,8,12^. On the other hand, the greatest deficiencies have been assigned to the domains of autonomy, waiting time, and choice^2,8,12^.

Considering the complexity of the Brazilian health system, especially regarding the execution of care practices focused on older adults, and the need to assess these services for the search of new proposals for planning and interventions in health, this study has aimed to analyze the quality of the outpatient care for older adults, using as a parameter the responsiveness of the service.

## METHODS

This is a descriptive cross-sectional study with a quantitative approach, carried out in a reference unit of the Brazilian Unified Health System (SUS) for the North and Northeast regions of the country. Located in Recife, State of Pernambuco, this institution treats approximately 67,000 older adults per year in appointments that offer clinical medical services and several specialties^a^.

The sample was probability with 385 older adults, using the total number of services provided in the institution in 2013. We took into consideration a frequency of 50.0% for the studied events (in order to maximize sample size), an absolute precision of 5.0 percentage points, and a 95.0% confidence level. The calculation of the sample was done using the program EpiInfo, version 6.04. The participants of this research were users who sought outpatient medical care, aged 60 years or more, who agreed to participate in the study.

The selection of individuals was carried out in the waiting room of the outpatient clinics, by order of arrival of the users. Considering the average care of 50 patients who could participate in the study, per day, we defined working with five patients per shift, using the calculation of “sampling interval”. A simple random drawing was carried out for the values from one to five to determine the older adults to be interviewed. The interviews took place in two shifts and were carried out taking into account the previous experiences of the older adults with the service.

We excluded from the sample the older adults who showed impossibility of communication, incoherence of speech or disorientation, those without a companion in cases in which patients did not know how to sign their name, and the first-time patients of the clinic. The identification of the older adults who presented the exclusion criteria of “impossibility of communication” and “incoherence of speech or disorientation” was based on the inability of the interviewee to answer questions about socioeconomic and demographic characteristics. In this way, the selection proceeded until we reached the sample defined initially.

The information was collected between March and July 2014 with an interview during the usual service of the clinic, without compromising the operation of the service. For the collection of information on the socioeconomic profile and self-assessment of health, we used closed questions of the Brazil Old Age Schedule (BOAS)^27^.

In the first part of the instrument, the following socioeconomic variables were considered: gender (female; male); race (white; brown; yellow; black; indigenous); age group (60 to 65 years; 66 to 70 years; ≥ 70 years); education level (no education; 1 to 4 years of study; 5 to 8 years of study; ≥ 9 years of study); monthly income (less than one minimum wage; equal to one minimum wage; more than one minimum wage; ignored); belief or religion (Catholic; Protestant; Spiritist; ignored); marital status (married or common-law marriage; widowed; divorced; never married or single); occupation (employed; unemployed; independent contractor; stay-at-home; retired; other).

For data on service responsiveness, we used the questionnaire of the Multi-Country Survey Study^b^, developed and validated by the WHO. This instrument allows the assessment of eight domains of responsiveness on aspects related to the environment where patients receive care at the outpatient level and the way how they are treated. These aspects are relevant for all types of health care, including individual and collective health services. Such domains can be understood as^28^:

1. Dignity: the state of being worthy or esteemed. It refers to the respectful care and physical examinations carried out in a reserved place.
2. Autonomy: the right of patients to participate in the decision-making about their own care and treatment if they wish. The health professional must ask permission before carrying out treatment or examining the patient.
3. Confidentiality: guaranteed confidentiality of the medical history of the patient and the conversations with the health professionals, in addition to the safe storage of medical records.
4. Communication: right to an understandable communication. The professional must carefully listen to patients, clearly explain their doubts, and ensure time for questions.
5. Fast service: health needs must be treated in a short period of time so as not to cause undue discomfort or distress. Furthermore, this implies short waiting time for appointments, as well as examinations and surgeries.
6. Support for social integration: health services must ensure access to social networks before, during, and after care. This means the supply of food to family members, freedom of religious practices, etc. This domain refers to the cases of hospitalization, not being used for research at the outpatient level.
7. Quality of physical facilities: right to the access to appropriate physical facilities. This considers the reasonable levels of comfort and environments conducive to the well-being of the user. In this way, services must offer ventilated waiting room and clean chairs and sectors (including the hygiene of toilets).
8. Choice: the patient must have the opportunity to choose the health professional who will carry out the appointment and be able to choose to be treated in another sector or institution.

For this study, we excluded the issues relating to the support for social integration, since they are restricted to hospitalization. Considering the regional variations, the research instrument was subjected to a pre-test with fifty users of the service, aiming at a better understanding by the target audience.

The approach advocated by the WHO is based on case studies and assessment of the responsiveness of health services. This type of study usually investigates the frequency that a given event occurs. Given this, the negative perceptions or those considered as a “problem” are defined as the percentage of persons who replied “never” or “sometimes”^28^. On the other hand, responsiveness considered as “good” or “adequate” refers to the percentage of persons who replied “always”. Additionally, interviewees were asked to indicate which domain they perceived as the most important for a quality care among the seven assessed.

The data were typed, with double entry, in the software EpiInfo, version 3.3.2. Pearson correlation test and Fisher’s test were used to verify if there was a relationship between the socioeconomic variables and adequate responsiveness. The results that showed significant association at the 95% level were presented in table, with their respective absolute and relative frequencies.

This study was approved by the Ethics Committee of the Universidade Federal do Pernambuco (Opinion 521,886/14), in accordance with resolution 466/12 of the National Health Council. All users who agreed to participate in the study signed the informed consent.

## RESULTS

We approached 396 older adults, of which 385 were interviewed and 11 (2.78%) refused to participate in the study.

Regarding the demographic profile of the sample, most were female (58%), white (62%), Catholic (61%), urban residents (92%), retired (75%), and with no dependents (75%). The highest prevalence concerns those aged between 60 and 65 years (42%). Among the others, 28% were aged between 66 and 70 years, and 30% were 70 years or older.

As for education level, 12% have never attended school, 40% had between one and four years of study, 22% studied five to eight years, and 26% reported nine or more years of study.

In relation to income, more than half received minimum wage (53%). Approximately 8% received less than minimum wage, 30% had monthly income greater than one minimum wage, and 9% preferred not to answer this question.

In addition, 25% of the older adults self-assessed their health as very good or good, 60% as regular, and 15% as poor or very poor.

Regarding the perception of the service provided by the unit, 57% considered it better than imagined, 6% thought that the service received was worse than expected, and 37% found that the service was within what they expected to receive. Nevertheless, most (98%) older adults stated that they recommend or would recommend the service to friends or relatives.

Among the domains studied, dignity, confidentiality, and communication reached the highest level of adequate responsiveness. On the other hand, freedom of choice and fast service received the greatest restrictions (Table 1).

**Table 1:** Percentage of adequate responsiveness in relation to the domains studied.

Domain	Aspect	Adequate responsiveness (%)
Choice	Freedom of choice	24.4
Autonomy	Participation in treatment decisions	49.7
Confidentiality	Confidentiality of information	91.3
Dignity	Respectful treatment by health professionals	97.9
	Respectful treatment by receptionists	75.2
	Respected privacy	87.9
Communication	Adequate listening by professionals	91.3
	Clarity in explanations	90.1
	Availability of time for questions or clarifications	84.2
Physical facilities	Cleanliness of the waiting room	90.0
	Cleanliness of toilets	46.3
	Ventilation of waiting room	50.0
	Availability of chairs	56.1
Fast service	Short waiting time for appointments, exams, and surgeries	22.0

Communication was reported as the most important domain for a quality care (41.2%), followed by dignity (39.2%), and fast service (11.5%) (Figure).

**Figure:**
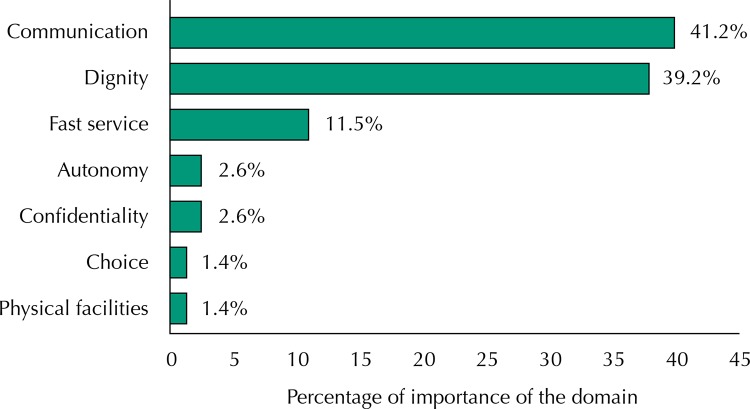
Distribution of domains of responsiveness regarding the percentage of importance.

Participation in decision-making regarding treatment (autonomy) was significantly lower (p = 0.018) among the older adults who did not attend school (Table 2). In a similar way, race influenced the type of assistance provided by health professionals who work in the service assessed (Table 3).

**Table 2:** Association* between adequate responsiveness regarding the domain of autonomy and the variable of education.

Education level	Autonomy (% of adequate responsiveness)
No education	9.6
1 to 4 years of study	37.1
5 to 8 years of study	19.8
≥ 9 years of study	33.5

* p = 0.018, Pearson’s Chi-square test.

**Table 3:** Association between adequate responsiveness (AR) regarding the aspects of privacy and adequate explanation and the variable of race.

Race	Adequate explanation		Privacy	
	
	AR (%)	p	AR (%)	p
White	65.3	0.027*	65.2	0.028*
Brown	20.8		20.9	
Black	13.9		13.9	

* Pearson’s Chi-square test, significant association at the 95% level.

The older adults that self-reported as black receive a lower quality of care regarding clear explanation and respected privacy in the appointment, when compared to users of any other race.

## DISCUSSION

The accelerated aging of the population has brought major changes in the profile of illness and resulted in increased demand for specialized services in this area^12,21^. Given this, the search for quality care provided by health services for older adults is currently considered a technical and social imperative.

The assessment of the responsiveness of health services in relation to the legitimate expectations of users is recognized as an important part of the performance of health systems^28^. However, little attention has been given to the population of older adults regarding this type of assessment^12^. Diógenes et al.^4^ confirm the importance of including this age group in service evaluation studies, since it would be a mistake to assume that all patients have the same needs and expectations.

The quality of the health care received by less educated older adults is inferior when compared to those who have a higher level of education^18^, resulting in a social inequality in the provision of such services. In a similar way, this study observed that older adults with no education had less opportunity to participate in decisions on their treatment in relation to the educated individuals.

When studying the consequences from this, Lima et al.^12^ have shown that older adults who did not have the right to exercise their autonomy report that “the doctor is in charge” and that if they did not “obey his order” they could even “lose the service.” This shows the passivity of users in relation to their case because of fear of losing the service, and it implies a real depersonalization and imposition of rules, disregarding the autonomy and the decision of the patient.

Although we still do not have concrete evidence about the possible factors that cause this type of attitude by health professionals, it is evident the need to plan and implement strategies aimed at the humanization of the care in order to ensure user participation as an active subject in the process of care. After all, the compliance and success of any proposed therapy are closely linked to the special service, based on trust and the respect to the wishes of users^25^.

Nevertheless, the autonomy of the patient is an ethical component of the care that needs to be constantly sought and encouraged regardless of the education level^3^. Under this perspective, researchers point out that contemporary medicine should favor the participation of the patients, their family members, and health professionals in the decision making to achieve the democratization of the doctor-patient relationship^23^.

In relation to fast service, the WHO^28^ has found that this domain is considered the most important by users of health services. In this study, this domain received the third highest score in importance (Figure). However, its performance was the worst among those assessed, being less than what was expected by the older adults (22%). In fact, the literature shows constant dissatisfaction of the users of public services regarding waiting time, which ratifies the need to develop strategies that provide a faster and more effective health care^1,20^.

It is evident the crisis in health systems because of the incongruity between the current epidemiological profile with predominance of chronic conditions and health services primarily directed to work in acute conditions and acute exacerbations of chronic diseases, organized, mostly, in a fragmentary way. These fragmented systems are unable to offer a continuous care to the population, resulting in delay in the service and low resolutiveness^17^.

Considering these aspects, Integrated Health Networks aim at the integration of health services in order to provide effective and consistent care in relation to the needs of users. Studies have shown that the action of services in integrated networks can improve clinical quality, health outcomes, and satisfaction of users, in addition to reducing the waiting time for the service and the costs of health care systems^17,22^.

Although this study found no significant association between education level and fast service (p = 0.191), a previous study^14^ has found greater time for the scheduling of appointments among the older adults who had no education. The results observed in this research may have been influenced by the low level of education prevalent among users of the institution assessed.

In relation to the variable race, the older adults who self-reported as black had less positive perception in relation to the respect for their privacy during the care (p = 0.028) and the explanations received from health professionals (p = 0.027) during the appointment. In this regard, Souza et al.^24^ had already observed that race is a limiting factor in the access to dental services for older adults, since black individuals had greater difficulty in using it.

In a health system that is intended to be universal and equitable, it is unacceptable that race differences interfere in aspects related to the care received. According to Lenardt et al.^11^, dignity for older adults must be inalienable and free from any conditioning bias that someone may wish to impose. As such, health professionals should provide a respectful care to older adults regardless of any other adjective characteristic.

It is important to note that communication with patients has a therapeutic function and it is considered an essential part in the process of care^11^. According to the opinion of the interviewees in this study, the professionals promote a relationship of dialogue with older adults, providing adequate listening and clear explanations regarding their health and/or treatment situation. This favors the link of the binomial user-health service, optimizing the process of care, as it gives professionals better knowledge on the issues inherent in the health situation of the patient, as well as the priorities of each case^8,12^.

Consistent with Lima et al.^12^, the choice of health professionals is among the domains that received the worst assessments by the older adults (24.4%). This is due to the reduced possibility of choosing the provider of services in public health units^2^.

This investigation found that the domains of dignity and confidentiality reached the highest level of adequate responsiveness in the institution assessed. These results corroborate the data from the WHO, which show that patients^28^ tend to report a good responsiveness for dignity and confidentiality in the assessment of outpatient services.

Lima et al.^12^, when assessing different public health services, have noted that most users were confident in care providers to ensure the confidentiality of their personal information. It is noteworthy that confidentiality is an essential requirement in the conduct of health professionals, and it is considered a right of the patient, as well as an ethical responsibility of care providers^19^.

However, there are reports in the literature related to services in which privacy in appointments was identified by users as the biggest flaw in the service provided. Among the complaints, we can point out that there is no appropriate place to change clothes to carry out exams, as well as access of other health professionals into the office, during the appointment, without the permission of the patient^9^. In these cases, it is evident the need for readjustment of the physical space of the health care facilities and the awareness of professionals regarding this component of the humanization of care.

With the exception of the cleanliness of the waiting room, the other aspects related to physical facilities were assessed as inadequate by almost half of the older adults interviewed. We highlight that the complaints of users are recurrent in the literature about the poor conditions of infrastructure and cleanliness of public health services^16^. The improvement of these aspects, most of the time, does not depend on large investments in financial and technological resources^9^ This situation refers to the context of organizational management and culture in which users are seen as low income customers served by the public health system and devoid of priority, in detriment to the spaces dominated by higher income customers^9^.

Furthermore on the responsiveness of health services, there is evidence that poor persons tend to mention worse responsiveness for the domains of fast service, dignity, communication, and choice^28^. This study did not identify the influence of such factors in the assessment of the unit researched. This investigation was carried out in an institution that works with the public network and serves, mostly, low income patients, favoring homogeneity of the sample, thus explaining this contradiction.

Although the interviewees point out some aspects that could be improved, most recommend or would recommend the service. Similar results have been found by Albuquerque^1^, who has assigned the high percentage of approval as a possible result of the confidence on the service received and the results achieved.

This study had as its main strength the ability to provide older adults with the opportunity to assess the quality of the service aimed at them. As possible limitations, we can mention that the study was carried out in the health unit and we excluded users who could not communicate and had incoherence of speech or disorientation. Some researchers indicate the importance of considering gratitude bias, as, in some cases, patients give a false positive assessment of the care for fear of losing the right to the service^26^. The influence of this type of bias may have been minimized from the clarification of the objectives of the research by the interviewer, ensuring the confidentiality of the information provided.

## CONCLUSIONS

The results of this study suggest that health professionals of the unit researched are working in providing a humanized care for older adults, with a view to proper communication and confidentiality of personal information reported in the appointment.

However, the service, regarding privacy and the explanations received during the appointment, was influenced by race, so that black older adults had a less positive perception about these aspects in relation to the other patients. This fact confirms the importance of the discussion on human rights and the fight for an egalitarian public health system.

In addition, it was evident the importance of considering patients as active subjects in discussions regarding their treatment, so as to ensure the autonomy of the older adults in the decisions inherent in their ongoing care.

It is worth noting that the freedom to choose the provider of the service and fast service were the domains that received the worst assessments. Furthermore, physical facilities were assessed as worse than expected by the older adults. These findings reinforce the need for investments aimed at providing an adequate proportion between demand and supply and the physical structure of the units for the specific needs of this age group.

Under this perspective, it is necessary to re-establish the coherence between the health situation, with a predominance of chronic conditions, and the action of health services, which are currently primarily directed to acute conditions and acute exacerbations of chronic diseases in a fragmentary way. To this end, Integrated Health Networks can offer a condition structurally more suited to perform integrated care and rationalize costs in order to optimize and redirect the use of resources to priority areas.

Given the above, public authorities and managers must be in line with the need for a health service that serves the population of older adults, considering its peculiarities.

## Footnotes

a Instituto de Medicina Integral Professor Fernando Figueira, Departamento de Contas Médicas. Recife: IMIP; 2013.

b World Health Organization. Multi-Country Survey Study. Brief 60min Questionnaire (Spanish). Geneva: HO [cited 2017 Feb 21]. Available from: www.who.int/responsiveness/surveys/en/
